# Associations between maternal health literacy, neonatal health and breastfeeding outcomes in the early postpartum period

**DOI:** 10.18332/ejm/170161

**Published:** 2023-10-03

**Authors:** Alma Gaupšienė, Aistė Vainauskaitė, Jekaterina Baglajeva, Rimantas Stukas, Diana Ramašauskaitė, Virginija Paliulytė, Natalja Istomina

**Affiliations:** 1 Department of Public Health of Institute of Health Sciences of Medical Faculty of Vilnius University, Vilnius, Lithuania; 2 Department of Nursing of Institute of Health Sciences of Medical Faculty of Vilnius University, Vilnius, Lithuania; 3 Center of Obstetrics and Gynecology of Institute of Clinical Medicine of Medical Faculty of Vilnius University, Vilnius, Lithuania

**Keywords:** health literacy, antenatal health, newborn health, breastfeeding

## Abstract

**INTRODUCTION:**

Maternal health literacy is a social skill that is relevant to successful postnatal newborn adaptation, neonatal feeding, and neonatal health outcomes, given the importance of maternal health literacy in newborn healthcare. The study aims to identify and assess the associations between maternal health literacy, neonatal health, and breastfeeding outcomes during the early postpartum period.

**METHODS:**

Five hundred women who gave birth to full-term newborns at Vilnius University Hospital were invited to the study from 1 May to 30 September 2022. The 47 questions of the European Health Literacy Questionnaire (HLS-EU-Q47) were used to assess maternal health literacy on days 2 and 3 after birth. Each subject’s health literacy indices were divided into four categories: inadequate, problematic, sufficient, and excellent. The neonatal health indicators were birth weight and height, along with the APGAR score and the outcomes of feeding either exclusively with breast milk or with adapted formula in addition to breastfeeding.

**RESULTS:**

Most women who participated in the survey had insufficient or problematic health literacy (69%). The study showed that women’s higher health literacy is associated with a lower risk of obesity, a healthier diet, regular physical activity, and a higher birth weight and height of their newborns (p<0.05). Mothers with inadequate/problematic health literacy were more likely to feed their newborns with adapted formula in addition to breastfeeding.

**CONCLUSIONS:**

Women’s health literacy is a factor that affects women’s healthy lifestyle choices before and during pregnancy and is significant for newborns’ health indicators.

## INTRODUCTION

Maternal health literacy is the cognitive and social skills relevant to access, understand, evaluate, and apply health information and services in ways that promote and support maternal and newborn health^1^. Health literacy skills determine the ability of mothers to assess their health status, make wise health decisions, and contact a healthcare institution in a timely manner^2,3^. The neonatal period (first 28 days of life) is vulnerable due to an underdeveloped immune system^4^. According to the World Health Organization (WHO), in 2020, 2.4 million newborns worldwide died in the first month of life. Around a third of all deaths occurred on the first day after birth, and nearly three-quarters in the first week of life^5^. The European Union’s (EU) lowest neonatal death rate in 2020 was 1.2/1000 in Norway, compared to 1.7/1000 live births in Lithuania^6^. Neonatal mortality is an important indicator for assessing the accessibility of the healthcare system, the quality of service provision, maternal health literacy and the ability to apply it appropriately to care for their own and their neonate’s health^5,6^.

Given the rising importance of health literacy in improving the health status of newborns, it is crucial to analyze the effect of maternal health literacy on breastfeeding behavior. Breastfeeding protects infants from infections, improves cognitive development, and reduces the development of obesity and chronic diseases^7^. Many factors, such as breastfeeding problems or the mother’s education and health literacy, can influence a woman’s decision not to breastfeed, potentially endangering the development and health of her newborn^8,9^.

Thus, to improve newborn health outcomes, it is essential to assess maternal health literacy, pinpoint the connections between neonatal health factors and breastfeeding outcomes, and emphasize maternal health literacy as a tool to improve neonatal health, encourage breastfeeding, and even lower neonatal mortality. The study aims to identify and assess the associations between maternal health literacy, neonatal health, and breastfeeding outcomes in the early postpartum period.

## METHODS

Women who gave birth to full-term (from 37+0 to 41+6 weeks) newborns were invited to participate in the study. The study excluded women aged <18 years and those who declined to participate in the survey. During the study, mothers’ health literacy was assessed by conducting a questionnaire survey on days 2 and 3 after birth (in the hospital). A total of 508 questionnaires were collected, of which 8 were rejected (not used for data analysis) since they were not filled in properly. The research was conducted at the Center of Obstetrics and Gynecology at Vilnius University Hospital from 1 May to 30 September 2022. The study maintained privacy, confidentiality, and anonymity, and the participants participated voluntarily and were free to discontinue participation at any time.

The 47 questions of the European Health Literacy Questionnaire (HLS-EU-Q47; freely available) were used to assess maternal health literacy (questions 1–47). Health literacy was evaluated in three domains: healthcare (questions 1–16), disease prevention (questions 7–31), health promotion (questions 32–47), and four stages of processing relevant information: to obtain (questions 1–4, 17–20, 32–36), understand (questions 5–8, 21–23, 37–40), evaluate (questions 9–12, 24–28, 41–43), and apply (questions 13–16, 29–31, 44–47)^10^.

A 4-point Likert scale was chosen to measure health literacy: 1=very difficult, 2=difficult, 3=easy, and 4=very easy, with the option to choose 5=‘I don’t know’ (this answer is not included in the summative health literacy assessment). Using the scores (from 1 to 4) of the answers to 47 questions, we calculated a general health literacy index, literacy indices for three health domains, and four indices of processing relevant health information for all subjects. Response scores were converted to a range from 0 to 50 to make comparisons clearer. We divided the health literacy indices calculated for each subject into four categories: 0–25, inadequate; 26–33, problematic; 34–42, sufficient; and 43–50, excellent^10^.

The chosen neonatal health indicators were birth weight, height, and the APGAR score (1 and 5 minutes after birth). We also recorded the outcomes of feeding either exclusively with breast milk or adapted formula in addition to breastfeeding on days 2 and 3 after birth. Neonatal weight and height were assessed using the WHO neonatal growth standards according to the percentiles^11^. Maternal weight gain throughout pregnancy was assessed as an additional factor in the analysis of newborns’ birth weight and height data following the guidelines of the Institute of Medicine (IOM)^12^. Moreover, we looked at the mother’s physical activity patterns before pregnancy, her nutrition, and her body mass index (ΒΜΙ, kg/m^2^) before and throughout pregnancy as potential influences on the newborn’s health. A woman’s number of births as a factor that might affect maternal health literacy (whether she is primiparous or multiparous) was also considered.

### Statistical analysis

Study data were analyzed with SPSS 23 software. The Shapiro-Wilk test was used to determine the normality of the compiled study data. For comparing two different study groups, Student’s t-test was used for independent samples; more than two variance analysis (one-way ANOVA) criteria were used for independent samples; and repeated measures ANOVA was used to compare the estimates of health literacy aspects of the same subjects. The chi-squared test was used to compare the distribution of percentage frequencies in the groups of subjects. To evaluate the linear dependence relationships between mothers’ health literacy and other numerical factors (newborn height, weight, and weight gain during pregnancy), Pearson or Spearman correlation analysis was applied. The calculated difference in the groups of subjects or the established relationship of dependence was considered statistically significant for p<0.05.

## RESULTS

Based on the calculated health literacy indices, women were divided into four groups: inadequate (16.4%; n=82), problematic (51.6%; n=258), sufficient (26%; n=130), and excellent (6%; n=30). According to the Student’s t-test results, all four health literacy indices of primiparous women were not significantly different from those of multiparous women (t=0.593; p>0.05). However, when their health information processing indices were compared, it became clear that multiparous women had much higher indices than primiparous women (Table 1). It was also shown that the health literacy indices of mothers who fed their newborn exclusively with their own milk and mothers who fed both with their own milk and adapted formula did not differ statistically significantly (χ^2^=1287; df=1; p>0.05). Participants who fit into the category of sufficient or excellent health literacy, based on the health promotion index, were approximately 1.2 times more likely to supplement nursing with the use of adapted formula (Table 2). The APGAR scale scores of neonates and mothers’ health literacy indices did not show any statistically significant correlations. The correlation coefficients revealed a weak, statistically significant association between the mother’s all four health literacy indices, her ability to obtain, understand, evaluate, and apply information, and her newborn’s height, with the newborn’s height increasing as the indices increased (p<0.01). Furthermore, higher birth weight was found to be associated with higher indices of maternal disease prevention and understanding of health information (p<0.01, p<0.05, respectively).

**Table 1:** Comparison of health information processing indices for primiparous and multiparous women, Lithuania (N=500)

*Health literacy indices*	*First birth*	*n*	*Mean*	*SD*	*t*	*df*	*p*
Ability to obtain information	Yes	205	30.13	7.94	0.625	462	0.532
	No	295	29.66	8.67			
Ability to understand information	Yes	205	33.45	6.53	0.418	498	0.676
	No	295	33.20	6.60			
Ability to evaluate information	Yes	205	28.72	6.97	-2.400	498	**0.017**
	No	295	30.22	6.82			
Ability to apply information	Yes	205	31.40	5.69	0.465	498	0.642
	No	295	31.15	5.77			

**Table 2:** Distribution of mothers with different health literacy levels according to their newborn’s feeding, Lithuania (N=500)

*Health literacy indices*	*Health literacy level*	*Breastfeeding n (%)*	*Breastfeeding + AF n (%)*	*Chi-squared test of independence*
Healthcare	IP	144 (53.3)	126 (46.7)	χ^2^=1.287 df=1 p=0.257
	SE	106 (48.2)	114 (51.8)	
Disease prevention	IP	155 (52.7)	139 (47.3)	χ^2^=0.851 df=1 p=0.356
	SE	95 (48.5)	101 (51.5)	
Health promotion	IP	179 (54.6)	149 (45.4)	**χ^2^=5.011 df=1 p=0.025**
	SE	71 (43.8)	91 (56.2)	
General health	IP	179 (53.9)	153 (46.1)	χ^2^=3.454 df=1 p=0.063
	SE	71 (44.9)	87 (55.1)	

AF: adapted formula. IP: inadequate/problematic health literacy. SE: sufficient/excellent health literacy.

Subjects who, according to the healthcare index, had sufficient/excellent health literacy (compared to those with inadequate/problematic literacy) were approximately two times more likely to give birth to overweight newborns (>97th percentile) (Table 3).

**Table 3:** Distribution of mothers with different health literacy according to their newborn weight, Lithuania (N=500)

*Health literacy indices*	*Health literacy level*	*Normal weight n (%)*	*Overweight n (%)*	*Chi-squared test of independence*
Healthcare	IP	249 (95.4)	12 (4.6)	**χ^2^=6.364 df=1 p=0.012**
	SE	193 (89.4)	23 (10.6)	
Disease prevention	IP	268 (93.7)	18 (6.3)	χ^2^=1.145 df=1 p=0.285
	SE	174 (91.1)	17 (8.9)	
Health promotion	IP	300 (93.8)	20 (6.3)	χ^2^=1.691 df=1 p=0.193
	SE	142 (90.4)	15 (9.6)	
General health	IP	302 (93.8)	20 (6.2)	χ^2^=1.849 df=1 p=0.174
	SE	140 (90.3)	15 (9.7)	

IP: inadequate/problematic health literacy. SE: sufficient/excellent health literacy.

Except for the healthcare index, subjects who scored sufficient or excellent in all other health literacy domains were 1.5 times less likely to be classified as women who gained too much weight during pregnancy, according to the guidelines published by the IOM (Table 4)^12^. Results revealed that mothers who belong to the categories of sufficient or excellent health literacy, according to all three health literacy indices and the general health literacy index, were notably less likely to be overweight or obese before becoming pregnant (p<0.01). Higher indices across all three health domains and in general health literacy were also associated with mothers snacking less often during pregnancy (p<0.05), consuming more vegetables before and during pregnancy (p<0.001) and consuming more fruit before and during pregnancy (p<0.001). Subjects with sufficient/excellent health literacy, according to the disease prevention, health promotion, and general health literacy indices, were approximately twice as likely to report exercising before pregnancy (p<0.001).

**Table 4:** Distribution of mothers with different health literacy by weight gain during pregnancy, according to guidelines published by the IOM, Lithuania (N=500)

*Health literacy indices*	*Health literacy level*	*Too little weight gain n (%)*	*Normal weight gain n (%)*	*Too much weight gain n (%)*	*Chi-squared test of independence*
Healthcare	IP	57 (21.8)	62 (23.7)	143 (54.6)	χ^2^=1.992 df=2 p=0.369
	SE	45 (20.9)	63 (29.3)	107 (49.8)	
Disease prevention	IP	48 (16.6)	70 (24.2)	171 (59.2)	**χ^2^=15.310 df=2 p<0.001**
	SE	54 (28.7)	55 (29.3)	79 (42.0)	
Health promotion	IP	60 (18.6)	80 (24.8)	183 (56.7)	**χ^2^=7.918 df=2 p=0.019**
	SE	42 (27.3)	45 (29.2)	67 (43.5)	
General health	IP	61 (18.9)	78 (24.2)	183 (56.8)	**χ^2^=7.939 df=2 p=0.019**
	SE	41 (26.5)	47 (30.3)	67 (43.2)	

IOM: Institute of Medicine. IP: inadequate/problematic health literacy. SE: sufficient/excellent health literacy.

## DISCUSSION

This study aimed to determine if there is a relationship between maternal health literacy and the health of their newborns, as well as breastfeeding outcomes. Firstly, we analyzed the mother’s BMI, her lifestyle habits prior to and throughout pregnancy, as well as the number of births, since these factors may have affected the health of the mother’s unborn child, independently of the mother’s level of health literacy. We discovered that women who have given birth more than once are substantially better at evaluating health information. As a result, they provide better care for their newborns^13^. However, it should be mentioned that the general health literacy index did not differ statistically significantly between these groups.

Maternal weight before pregnancy is a significant factor since maternal obesity raises the risk of preterm birth, structural fetal anomalies, high gestational age, and perinatal death^14^. According to all health literacy indices, mothers with sufficient or excellent health literacy were much less likely to be overweight or obese prior to becoming pregnant, indicating that they are less likely to experience negative outcomes related to obesity. Neonatal low birth weight is more probable when a woman’s weight gain during pregnancy is insufficient^15^, whereas macrosomia, increased rates of cesarean section, and perinatal complications are more likely when weight gain is excessive^16^. Our study showed that women who had sufficient or excellent health literacy were less likely to have gained too much weight during pregnancy. Additionally, pregnant women with sufficient or excellent levels of health literacy across all literacy indices more often exercised before becoming pregnant, included more vegetables and fruit in their diets during pregnancy, and avoided frequent snacking. Thus, this group of women is better equipped to assess and use knowledge about healthy lifestyle choices that may help ensure their newborn’s health^17^.

While investigating the relationship between maternal health literacy and the newborn’s health status, we discovered that mothers with sufficient or excellent health literacy were about twice as likely to deliver heavier newborns. We believe women with sufficient or excellent health literacy pay closer attention to their dietary choices, study product information and nutrition labels, and consume enough calories. Chia et al.^18^ indicate in their study that mothers with healthy eating habits delivered babies with greater birth weight, while mothers with unhealthy eating patterns gave birth to babies with lower birth weight. Furthermore, it is likely that women with sufficient or excellent health literacy start planning their pregnancy in advance and begin taking vitamins earlier, especially folic acid, which has been linked to higher birth weight in past clinical trials^19^. In our research, we discovered that neonates are born taller as health literacy increases. However, it should be noted that we cannot completely rule out a genetic predisposition since the parents (mother and father) heights were not measured. We believe that women with sufficient or excellent health literacy make healthy food and vitamin choices, consume enough calories, and, as a consequence, provide adequate nutrition to their babies. As a result, their weight and height do not fall below the norm.

When analyzing the influence of maternal health literacy on the health of newborns via APGAR score, we found contradictory findings. Some claim that newborns of mothers with inadequate health literacy have a higher proportional risk of having a 5-minute APGAR score <4^20^. However, no statistically significant correlations between maternal health literacy and APGAR score were found in our study. Breast milk is the best option for infants since it helps establish their immunity and provides a variety of antibodies, enzymes, hormones, and balanced nutrients that affect the development of the digestive and nervous systems^21^. Most expecting mothers give birth in hospitals, where they receive active support and counseling regarding breastfeeding. This support encourages mothers to breastfeed their newborns, eliminating supplemental feeding with an adapted formula that is unnecessary^22^. However, only the mother makes the final decision about how her child is fed. Our findings indicate that women with sufficient or excellent health literacy in the health promotion index were almost two times more likely to feed their newborns with adapted formula in addition to their own milk. These findings suggest some potential causes after giving birth: some women are unprepared for motherhood, they have insecurity, a lack of self-confidence, and a need for attention or support from medical staff. As a result of excessive information reading, mothers decide to supplement breastfeeding with adapted formula since they fear their newborns will not get enough breast milk^7^. Promoting antenatal education or other informative educational activities is crucial since it allows women to obtain accurate information about pregnancy nutrition, physical activity, breastfeeding, and newborn care. Since this practice is not currently mandated, regulated, or compensated by health funds, it possibly contributes to a lack of health literacy among Lithuanian mothers.

### Limitations

Limitations of the study include the possibility of reporting bias and limited generalizability of the study findings to other settings. Further research is needed to collect additional information from women in other Lithuanian hospitals to represent the whole Lithuanian healthcare system.

## CONCLUSIONS

The findings of this study indicate that a woman’s health literacy is an influential factor that can have an immense effect not only on her lifestyle (dietary decisions, physical activity) but also on her newborn’s physical condition (height, weight) and breastfeeding behavior. This observed effect is partly positive: women with greater health literacy skills consume more fruits and vegetables, snack less frequently, engage in more physical activity, are less likely to be obese, and are less likely to give birth to short and underweight newborns. Nevertheless, women with higher health literacy are less likely to exclusively breastfeed their newborns. More actions should be taken to help women prepare for motherhood, improve public health literacy, and encourage healthy lifestyles, considering that the majority of the women surveyed had inadequate or problematic health literacy.

## Data Availability

The data supporting this research are available from the authors on reasonable request.
